# Ambulatory Follow-up and Outcomes Among Medicare Beneficiaries After Emergency Department Discharge

**DOI:** 10.1001/jamanetworkopen.2020.19878

**Published:** 2020-10-09

**Authors:** Michelle P. Lin, Ryan C. Burke, E. John Orav, Tynan H. Friend, Laura G. Burke

**Affiliations:** 1 Department of Emergency Medicine, Icahn School of Medicine at Mount Sinai, New York, New York; 2 Department of Emergency Medicine, Beth Israel Deaconess Medical Center, Harvard Medical School, Boston, Massachusetts; 3 Harvard Global Health Institute, Cambridge, Massachusetts; 4 Department of Biostatistics, Harvard T.H. Chan School of Public Health, Boston, Massachusetts; 5 Department of General Internal Medicine, Brigham and Women’s Hospital, Boston, Massachusetts; 6 Department of Health Policy and Management, Harvard T.H. Chan School of Public Health, Boston, Massachusetts

## Abstract

**Importance:**

Ambulatory follow-up care is frequently recommended after an emergency department (ED) visit. However, the frequency with which follow-up actually occurs and the degree to which follow-up is associated with postdischarge outcomes is unknown.

**Objectives:**

To examine the frequency and variation in ambulatory follow-up among Medicare beneficiaries discharged from US EDs and the association between ambulatory follow-up and postdischarge outcomes.

**Design, Setting, and Participants:**

This cohort study of 9 470 626 ED visits to 4728 US EDs among Medicare beneficiaries aged 65 and older from 2011 to 2016 who survived the ED visit and were discharged to home used Kaplan-Meier curves and proportional hazards regression. Data analysis was conducted from December 2019 to July 2020.

**Exposures:**

Ambulatory follow-up after discharge from the ED.

**Main Outcomes and Measures:**

Postdischarge mortality, subsequent ED visit, or inpatient hospitalization within 30 days of an index ED visit.

**Results:**

The study sample consisted of 9 470 626 index outpatient ED visits to 4684 EDs; most visits (5 776 501 [61.0%]) were among women, and the mean (SD) age of patients was 77.3 (8.4) years. In this sample, the cumulative incidence of ambulatory follow-up was 40.5% (3 822 133 patients) at 7 days and 70.8% (6 662 525 patients) at 30 days, after accounting for censoring and for mortality as a competing risk. Characteristics associated with lower rates of ambulatory follow-up included beneficiary Medicaid eligibility (hazard ratio [HR], 0.77; 95% CI, 0.77-0.78; *P* < .001), Black race (HR, 0.82; 95% CI, 0.81-0.83; *P* < .001), and treatment at a rural ED (HR, 0.75; 95% CI, 0.73-0.77; *P* < .001) in the multivariable regression model. Ambulatory follow-up was associated with lower risk of postdischarge mortality (HR, 0.49; 95% CI, 0.49-0.50; *P* < .001) but higher risk of subsequent inpatient hospitalization (HR, 1.22; 95% CI, 1.21-1.23; *P* < .001) and ED visits (HR, 1.01; 95% CI, 1.00-1.01; *P* < .001), adjusting for visit diagnosis, patient demographic characteristics, and chronic conditions.

**Conclusions and Relevance:**

In this cohort study of Medicare beneficiaries discharged from the ED, nearly 30% lacked ambulatory follow-up at 30 days, with variation in follow-up rates by patient and hospital characteristics. Having an ambulatory follow-up visit was associated with higher risk of subsequent hospitalization but lower risk of mortality. Ambulatory care access may be an important driver of clinical outcomes after an ED visit.

## Key Points

**Question:**

How often do Medicare beneficiaries have an ambulatory follow-up visit after discharge from the emergency department, and is ambulatory follow-up associated with postdischarge outcomes?

**Findings:**

In this cohort study of 9 470 626 emergency department discharges from 2011 to 2016, most patients had ambulatory follow-up within 30 days, with lower rates among Medicaid-eligible beneficiaries, Black beneficiaries, and those treated at rural emergency departments. Ambulatory follow-up was associated with a higher risk of subsequent hospitalization but a lower risk of 30-day mortality.

**Meaning:**

The findings of this study suggest that access to ambulatory care may be a key driver of outcomes among Medicare beneficiaries discharged from the emergency department.

## Teaser

This cohort study examines the frequency and variation in ambulatory follow-up among Medicare beneficiaries discharged from US emergency departments (EDs) and the association between ambulatory follow-up and postdischarge outcomes.

## Introduction

Nearly 1 in 5 US residents visit the emergency department (ED) every year.^1^ An increasing share of ED visits result in discharge home rather than inpatient hospitalization.^2^ However, among patients who are discharged from the ED, there is substantial variation in postdischarge outcomes.^3,4^ Among older adults in particular, the days after an ED discharge may be associated with an increased risk of hospitalization or unexpected death.^4,5^ Thus, improving care for patients discharged from the ED may be an important target for improving quality and outcomes.^2,3^

Timely follow-up care after ED discharge may improve outcomes by ensuring that the acute problem prompting the ED visit has not worsened and optimizing management of chronic diseases, if needed.^6,7,8,9,10,11^ However, there is limited evidence describing how frequently follow-up after ED discharge occurs or the degree to which ambulatory follow-up rates differ by patient or hospital characteristics.^12,13,14,15^ Health insurance can improve access to follow-up care but does not eliminate barriers to care, even among Medicare beneficiaries.^16,17,18^ Furthermore, federal agencies and national societies have endorsed timely follow-up care after ED discharge as an indicator of the quality of care, despite limited evidence regarding whether such follow-up care actually improves outcomes.^6,7,8,9,10,11,19,20^

Therefore, we used national Medicare data from 2011 to 2016 to examine the following questions. First, how often do Medicare beneficiaries discharged from the ED have an ambulatory follow-up visit within 7 and 30 days? Second, are certain patient or hospital characteristics associated with higher rates of ambulatory follow-up after ED discharge? Third, is ambulatory follow-up after ED discharge associated with differences in the postdischarge outcomes of mortality and subsequent ED or inpatient utilization?

## Methods

### Data Source

We examined a random 20% of Medicare beneficiaries from 2011 to 2016 who had an outpatient ED visit and were discharged home (eAppendix in the Supplement). We excluded 69 182 ED visits at nonacute care and federal hospitals (eFigure in the Supplement), those outside of the 50 states and the District of Columbia (50 836), and those without a diagnosis or whose principal diagnosis did not match a Clinical Classifications Software category (572 361).^21^ We further excluded visits resulting in discharge to a nursing or rehabilitation facility (680 064). We adhered to all Strengthening the Reporting of Observational Studies in Epidemiology (STROBE) reporting guideline for observational studies.^22^ The Harvard T.H. Chan School of Public Health institutional review board approved this study. Informed consent was waived because of the retrospective nature of the data.

### Patients

Beneficiaries aged 65 years or older who were continuously enrolled in fee-for-service Medicare were included. Beneficiary age, sex, race (based on self-report), Medicaid enrollment, and death date were obtained from the Medicare denominator file.^23^ Chronic conditions were classified according to the Centers for Medicare & Medicaid Services Chronic Conditions Warehouse categories.

### ED Visits

We aggregated principal diagnoses based on prior research into 38 clinically meaningful categories designed for studies of outcomes after ED visits (eAppendix in the Supplement).^21,24^ ED visits were identified from the Medicare inpatient and outpatient claims files. We also examined hospital size, teaching status, urban or rural location, ownership, and safety-net status from linked 2014 American Hospital Association annual survey and Medicare Impact Files (eAppendix in the Supplement).^25^ Hospitals in the top quartile of disproportionate share percentage were considered safety-net hospitals; all others were classified as non–safety net.^26^ We also identified hospital performance on 2 publicly reported quality measures, ie, Hospital Compare overall star rating and the proportion of patients who gave a high rating on the Hospital Consumer Assessment of Healthcare Providers and Systems patient experience survey.^27,28^

### Ambulatory Follow-up Visits

The primary exposure of interest was the number of days until ambulatory follow-up after ED discharge. We calculated daily event rates up to 90 days but focused on events within 30 days. We identified ambulatory follow-up visits from the Medicare Part B Carrier professional claims file, which were categorized according to Berenson-Eggers type of service codes and place of service codes to exclude visits to EDs and residential rehabilitation facilities. We identified the specialty of the provider associated with each follow-up visit (eAppendix in the Supplement).

### Outcomes

The primary outcomes were time to mortality, subsequent ED visit (all types, regardless of disposition), or inpatient hospitalization within 30 days of ED discharge. For these outcomes, time to ambulatory follow-up was treated as a time-varying covariate, ensuring that only those follow-up visits occurring before the respective outcomes were considered when assessing the association between follow-up and the risk of these outcomes. Once the follow-up visit occurred, that patient’s hazard of the outcome occurring changed to the degree indicated by the hazard ratio (HR) from the model. This change is relative to other patients (at the same time after their ED visits) who have not yet had a follow-up visit.

### Statistical Analysis

#### Ambulatory Follow-up After ED Discharge

A Kaplan-Meier curve was generated to estimate the time-dependent probability of ambulatory follow-up after ED discharge, treating death as a competing risk and 30 days as the censoring time. To examine the associations between patient and hospital characteristics and ambulatory follow-up, we initially compared patient characteristics (ie, age, sex, race, and Medicaid eligibility) and hospital characteristics (ie, size, region, teaching status, control, rural status, and safety-net status) between patients who did and did not have an ambulatory visit within 30 days. These comparisons were formalized in a proportional hazards regression model with time until ambulatory follow-up as the outcome, death as a competing risk, and 30 days as the censoring time. We included patient and hospital characteristics, visit year, principal visit diagnosis category, and beneficiary chronic conditions to adjust for differences in severity between patients that may be associated with timing of follow-up. Finally, to account for clustering of patients within hospitals, we used the analogue of generalized estimating equations to adjust for correlation between the within-hospital residuals using the sandwich estimate of the covariance matrix. To avoid multiple testing, results are presented as HRs and 95% CIs. All hypothesis tests were 2-sided, and statistical significance was set at *P* < .05. Analyses were performed in SAS version 9.4 (SAS Institute).

#### Association Between Ambulatory Follow-up After ED Discharge and Postdischarge Outcomes

To examine the association between ambulatory follow-up and postdischarge outcomes, we specified 3 separate Cox regression models for the outcomes of mortality, all subsequent ED visits, and inpatient hospitalizations after discharge from the index ED visit. For each model, our primary exposure was ambulatory follow-up as a time-varying covariate to avoid immortality bias (ie, potential bias because patients who had an ambulatory follow-up visit had to be alive at least that long).^29^ We incorporated the year of visit, principal visit diagnosis, beneficiary demographic characteristics, and beneficiary chronic conditions as covariates and accounted for hospital-level clustering by stratifying by hospital. Stratification allowed each hospital to have its own unique baseline hazard for ambulatory follow-up, much like fixed effects for hospitals in linear and logistic regression models, with the added benefit that the hazards were not assumed to be proportional between hospitals. All patients within the same hospital shared the same baseline hazard so that patients with ambulatory follow-up were compared only with patients without ambulatory follow-up in the same hospital. For the outcomes of subsequent ED visits and inpatient hospitalizations, we incorporated mortality as a competing risk. For the association of ambulatory follow-up with each of these 3 outcomes, we show *P* values, HRs, and confidence intervals. Because of multiple testing, *P* < .0167 denotes significance.

#### Sensitivity Analyses

We repeated our main models limiting follow-up to 7 days to check the robustness of the choice of censoring time as well as to qualitatively evaluate the proportional hazards assumption by checking whether the HR remained similar in early follow-up as in late follow-up. To assess potential differences by clinical condition, we repeated our main analyses separately for 10 clinical conditions with the largest magnitude risk of postdischarge mortality, defined by the regression coefficient for the outcome of mortality in our main Cox regression model. We also examined whether any patient-level associations between ambulatory follow-up and postdischarge events differed by hospital-level practice patterns and ambulatory care access. We first calculated hospital-level risk-adjusted rates of ambulatory follow-up using a linear probability model with follow-up rates as the outcome and adjusting for hospital random effects, principal diagnosis, and beneficiary characteristics. We then created 3 groups of hospitals based on their adjusted rates of ambulatory follow-up after ED discharge into high follow-up (top quartile), medium follow-up (middle 50%), and low follow-up hospitals (bottom quartile). We repeated our visit-level Cox regression models with ambulatory follow-up as a time-varying covariate as the primary exposure for each of the 3 postdischarge outcomes. We ran these models separately for high, medium, and low follow-up hospitals. Additionally, to examine whether rates of ambulatory follow-up were associated with other indicators of quality, we calculated the adjusted rates of each postdischarge outcome as well as the mean Hospital Compare overall star rating and proportion of patients giving a high rating of the Hospital Consumer Assessment of Healthcare Providers and Systems patient experience survey for each stratum of hospital follow-up.

## Results

### Ambulatory Follow-up After Discharge From the ED

**Figure 1.**
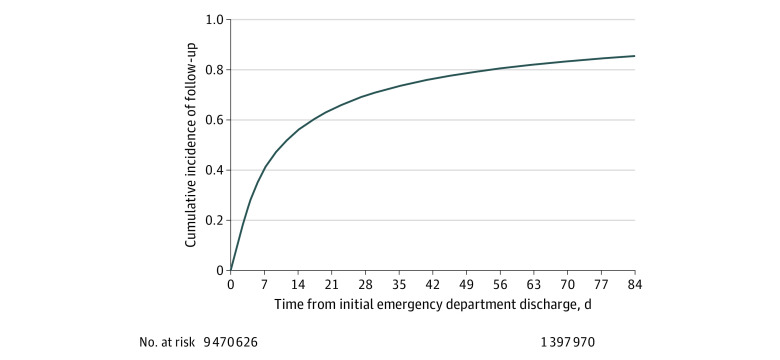
Time to Ambulatory Follow-up Among Medicare Beneficiaries Aged 65 Years and Older Treated and Discharged From the Emergency Department From 2011 to 2016 Median time to follow-up was approximately 10 days, with 40.4% of patients (3 822 133) having follow-up by 7 days, 70.8% (6 662 525) by 30 days, and 86.1% (8 059 974) by 90 days.

The study sample consisted of 9 470 626 index outpatient ED visits to 4728 EDs; most patients (61.0%) were women (Table 1), and the mean (SD) age was 77.3 (8.4) years. The cumulative incidence of ambulatory follow-up was 21.5% (2 037 280 patients) at 3 days, 40.5% (3 822 133 patients) at 7 days, 70.8% (6 662 525 patients) at 30 days, and 86.1% (8 059 974 patients) at 90 days, after accounting for censoring and mortality as a competing risk (Figure 1). The distribution of the specialty of the clinician at follow-up visit is shown in eTable 1 in the Supplement, with 2 827 688 of 6 662 525 visits (42.4%) of visits occurring among primary care specialties. Patient and hospital characteristics are shown in Table 1. Adjusted associations are presented in Table 2. Patient characteristics associated with lower hazard of postdischarge follow-up included beneficiary Medicaid eligibility (HR, 0.77; 95% CI, 0.77-0.78; *P* < .001) as well as Black race (HR, 0.82; 95% CI, 0.81-0.83; *P* < .001). Treatment at a rural ED was associated with a 25% lower hazard of follow-up (HR, 0.75; 95% CI, 0.73-0.77; *P* < .001) after accounting for patient and hospital characteristics and chronic conditions.

**Table 1.** Patient and Hospital Characteristics for ED Visits Among Medicare Beneficiaries From 2011 to 2016^a^

Characteristic	No. (%)		
	All ED visits (N = 9 470 626)	Visits with follow-up in 30 d	
		No (n = 2 725 341)^b^	Yes (n = 6 601 306)
**Patient characteristics**			
Age, y			
65-74	4 082 280 (43.1)	1 205 203 (44.2)	2 810 364 (42.6)
75-84	3 267 146 (34.5)	850 481 (31.2)	2 368 856 (35.9)
≥85	2 121 200 (22.4)	669 657 (24.6)	1 422 086 (21.5)
Sex			
Women	5 776 501 (61.0)	1 618 158 (59.4)	4 072 674 (61.7)
Men	3 694 124 (39.0)	1 107 183 (40.6)	2 528 631 (38.3)
Race/ethnicity			
White	7 883 933 (83.2)	2 192 244 (80.4)	5 572 191 (84.4)
Black	1 059 996 (11.2)	367 751 (13.5)	676 618 (10.3)
Hispanic	194 370 (2.1)	64 955 (2.4)	126 614 (1.9)
Other^c^	282 530 (3.0)	85 621 (3.1)	192 281 (2.9)
Missing	49 797 (0.5)	14 770 (0.5)	33 602 (0.5)
Medicaid eligible			
Yes	2 119 365 (22.4)	772 768 (28.4)	1 317 475 (20.0)
No	7 351 261 (77.6)	1 952 573 (71.7)	5 283 831 (80.0)
5 Most frequent principal diagnosis categories			
Other injuries	1 462 292 (15.5)	495 991 (18.2)	946 202 (14.3)
Disease of the musculoskeletal system	999 721 (10.6)	272 730 (10.0)	711 465 (10.8)
Minor injuries	731 871 (7.7)	179 423 (6.6)	541 529 (8.2)
Gastrointestinal system disease	631 705 (6.7)	186 194 (6.8)	436 105 (6.6)
Other symptoms	408 548 (4.3)	120 783 (4.4)	282 262 (4.3)
Missing	3992 (0.04)	719 (0.03)	1892 (0.03)
**Hospital characteristics**			
Size, No. of beds			
Small, 1-99	2 202 319 (23.3)	786 882 (28.9)	1 383 127 (21.0)
Medium, 100-399	5 025 977 (53.1)	1 344 495 (49.3)	3 604 495 (54.6)
Large, ≥400	2 133 234 (22.5)	560 091 (20.5)	1 540 156 (23.3)
Missing	109 096 (1.2)	33 873 (1.2)	73 528 (1.1)
Region			
Northeast	1 616 295 (17.1)	432 750 (15.9)	1 160 225 (17.6)
Midwest	2 142 256 (22.6)	638 092 (23.4)	1473061 (22.3)
South	3 869 165 (40.9)	1 110 967 (40.8)	2 698 910 (40.9)
West	1 733 814 (18.3)	509 659 (18.7)	1 195 582 (18.1)
Missing	109 096 (1.2)	33 873 (1.2)	73 528 (1.1)
Teaching status			
Major	931 479 (9.8)	247 566 (9.1)	669 319 (10.1)
Minor	2 883 941 (30.5)	775 429 (28.5)	2 064 487 (31.3)
Nonteaching	5 546 110 (58.6)	1668 473 (61.2)	3 793 972 (57.5)
Missing	109 096 (1.2)	33 873 (1.2)	73 528 (1.1)
Control type			
For profit	1 355 092 (14.3)	380 902 (14.0)	953 093 (14.4)
Nonprofit	6 692 762 (70.7)	1 861 508 (68.3)	4 729 714 (71.7)
Government, nonfederal	1 313 676 (13.9)	449 058 (16.5)	844 971 (12.8)
Missing	109 096 (1.2)	33 873 (1.2)	73 528 (1.1)
Urban or rural			
Rural	830 580 (8.8)	356 259 (13.1)	462 888 (7.0)
Urban	8 530 950(90.1)	2 335 209 (85.7)	6 064 890 (91.9)
Missing	109 096 (1.2)	33 873 (1.2)	73 528 (1.1)
Safety net			
Yes	1 740 115 (18.4)	543 145 (19.9)	1 170 888 (17.7)
No	7 730 511 (81.6)	2 182 196 (80.1)	5 430 418 (82.3)

Abbreviation: ED, emergency department.

^a^ 20% sample of visits among Medicare beneficiaries aged 65 years and older who were enrolled in traditional Medicare to the ED at US acute care hospitals in 2011 to 2016.

^b^ We present unadjusted patient and hospital characteristics among the subset of visits through December 2, 2016, to ensure a full 30 days of follow-up (n = 9 326 647).

^c^ This group includes individuals who self-reported race/ethnicity as Asian, Hispanic, North American Native, and other.

**Table 2.** Association Between Patient and Hospital Characteristics and 30-Day Ambulatory Follow-up Among Medicare Beneficiaries Aged 65 Years and Older Treated and Discharged From the ED From 2011 to 2016^a^

Characteristic	HR (95% CI)^b^
Year of ED visit	1.00 (0.999-1.001)
Age, y	0.997 (0.997-0.997)
Sex	
Women	1 [Reference]
Men	0.90 (0.898-0.904)
Race/ethnicity	
White	1 [Reference]
Black	0.82 (0.81-0.83)
Hispanic	0.96 (0.94-0.98)
Asian	1.07 (1.04-1.09)
North American Native	0.87 (0.84-0.90)
Other^c^	1.01 (0.99-1.03)
Unknown	1.00 (0.98-1.02)
Medicaid eligible	
No	1 [Reference]
Yes	0.77 (0.77-0.78)
Hospital size, No. of beds	
Large, ≥400	1 [Reference]
Small, 1-99	0.87 (0.85-0.89)
Medium, 100-399	0.99 (0.98-1.01)
Control type	
Nonprofit	1 [Reference]
For profit	1.00 (0.99-1.02)
Government, nonfederal	0.93 (0.92-0.95)
Teaching status	
Major	1 [Reference]
Minor	0.99 (0.97-1.01)
Nonteaching	0.98 (0.95-0.998)
Urban/rural	
Urban	1 [Reference]
Rural	0.75 (0.73-0.77)
Safety-net status	
No	1 [Reference]
Yes	0.94 (0.93-0.96)
Region	
Northeast	1 [Reference]
Midwest	0.96 (0.94-0.97)
South	1.01 (0.99-1.02)
West	1.01 (0.99-1.03)

Abbreviations: ED, emergency department; HR, hazard ratio.

^a^ Cox proportional hazards model with time to ambulatory follow-up as the outcome and beneficiary age, sex, race, and Medicaid eligibility as covariates.

^b^ The multivariable model incorporated year of the visit, principal diagnosis category, beneficiary demographic characteristics and chronic conditions, and hospital characteristics as covariates, including clustering by hospital in a single Cox regression model. Mortality was accounted for as a competing risk. An HR less than 1 indicates a longer time until follow-up visit.

^c^ This group includes individuals who self-reported race/ethnicity as Asian, Hispanic, North American Native, and other.

### Association Between Ambulatory Follow-up and 30-Day Postdischarge Mortality, Subsequent ED Visit, and Inpatient Hospitalization

**Figure 2.**
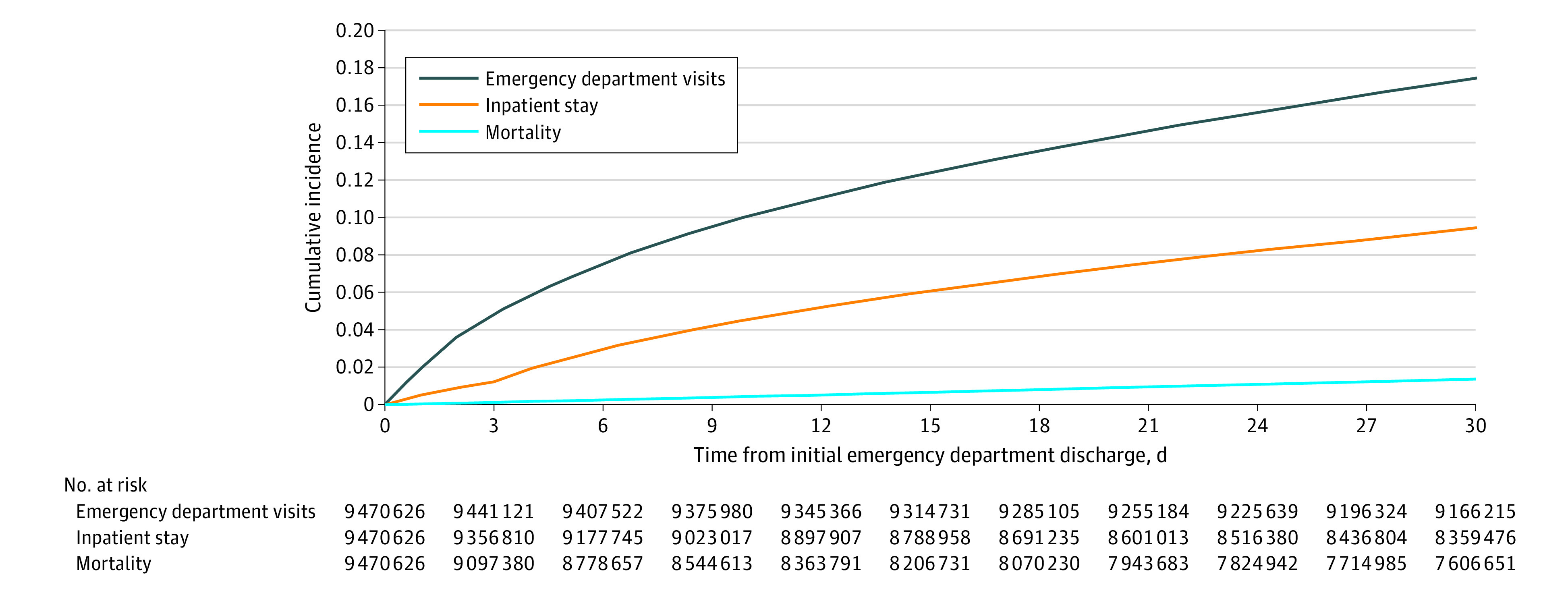
Rates of Postdischarge Events Among Medicare Beneficiaries Aged 65 Years and Older Treated and Discharged From the Emergency Department From 2011 to 2016 Kaplan-Meier curves were generated for each of the following outcomes after emergency department discharge: mortality, subsequent emergency department visit, and inpatient stay. The outcomes of emergency department visits and inpatient stay account for mortality as a competing risk in the survival analysis.

Our initial sample of 9 470 626 ED visits included 3 711 826 beneficiaries, of whom 127 412 died and 42 668 were censored for termination of enrollment during the study period. Among the sample of 6 662 525 ED visits ending in discharge, there were 1 640 598 return visits to the ED and 885 761 subsequent hospitalizations. The 30-day cumulative incidence for postdischarge outcomes was 1.4% for mortality, 17.4% for postdischarge ED visit, and 9.4% for postdischarge hospitalizations when accounting for censoring and mortality as a competing risk (Figure 2). Ambulatory follow-up after ED discharge was associated with 51% lower risk of mortality within 30 days of ED discharge compared with those with no follow-up visit (HR, 0.49; 95% CI, 0.49-0.50; *P* < .001) (Table 3). Ambulatory follow-up was associated with a 1% higher hazard of a subsequent ED visit within 30 days (HR, 1.01; 95% CI, 1.003-1.01; *P* < .001) and a 22% higher hazard of an inpatient stay (HR, 1.22; 95% CI, 1.21-1.23; *P* < .001). The associations between all covariates in the model and risk of postdischarge outcomes are presented in eTable 2 in the Supplement.

**Table 3.** Association Between Ambulatory Follow-up and Risk of 30-Day Postdischarge Mortality, Subsequent ED Visit, and Inpatient Hospitalization Among Medicare Beneficiaries Treated in the ED and Discharged From 2011 to 2016, Overall and Stratified by Hospital Follow-up Category^a^

Outcome	HR (95% CI)^b^	*P* value
**All visits**		
Mortality	0.49 (0.49-0.50)	<.001
Subsequent ED visit	1.010 (1.003-1.030)	<.001
Inpatient stay	1.22 (1.21-1.23)	<.001
**Visits to high follow-up hospitals**^**c**^		
Mortality	0.47 (0.46-0.48)	<.001
Subsequent ED visit	1.02 (1.01-1.02)	<.001
Inpatient stay	1.22 (1.20-1.25)	<.001
**Visits to medium follow-up hospitals**^**c**^		
Mortality	0.50 (0.49-0.51)	<.001
Subsequent ED visit	1.00 (0.99-1.00)	.82
Inpatient stay	1.22 (1.21-1.23)	<.001
**Visits to low follow-up hospitals**^**c**^		
Mortality	0.60 (0.58-0.63)	<.001
Subsequent ED visit	1.02 (1.01-1.03)	.001
Inpatient stay	1.22 (1.20-1.25)	<.001

Abbreviations: ED, emergency department; HR, hazard ratio.

^a^ Cox proportional hazards model with the time to each postdischarge event as the outcome and ambulatory follow-up as a time-varying covariate as the primary exposure. We incorporated beneficiary age, sex, race, and Medicaid eligibility, year of visit, principal diagnosis category, and beneficiary chronic conditions as covariates and accounted for clustering by hospital. For the outcomes of ED visits and inpatient stays, we also incorporated mortality as a competing risk.

^b^ An HR less than 1 indicates a longer time until the outcome event.

^c^ Three groups of hospitals were created based on their adjusted rates of ambulatory follow-up after ED discharge into high follow-up (top quartile), medium follow-up (middle 50%), and low follow-up (bottom quartile) hospitals. We repeated our main models separately for high, medium, and low follow-up hospitals.

### Sensitivity Analyses

The association between ambulatory follow-up and lower mortality and higher hospitalization risk was consistent after limiting the survival analysis to 7 days (eTable 3 in the Supplement). However, follow-up visits were associated with a 4% lower risk of a subsequent ED visit (HR, 0.96; 95% CI, 0.95-0.96; *P* < .001) at 7 days compared with a 1% higher risk at 30 days. When we repeated our analysis separately for 10 high-risk conditions with the greatest risk of postdischarge mortality, our findings were similar (eTable 4 in the Supplement), although we generally found higher rates of follow-up for these high-risk conditions compared with the overall sample and a greater magnitude of the association between ambulatory follow-up and lower postdischarge mortality.

The mean (SD) hospital-level rate of 30-day ambulatory follow-up was 65.1% (13.1%). Mean follow-up rates among hospitals in the bottom quartile, middle 50%, and highest quartile of ambulatory follow-up were 46.4%, 68.7%, and 76.9%, respectively. After repeating our analysis examining the association between ambulatory follow-up as a time-varying covariate and hazard of postdischarge events, there was again a consistent association between ambulatory follow-up and lower adjusted mortality. However, the magnitude of this association was greatest at hospitals with the highest follow-up rates (HR, 0.47; 95% CI, 0.46-0.48) compared with medium (HR, 0.50; 95% CI, 0.49-0.51) and low (HR, 0.60; 95% CI, 0.58-0.63) follow-up hospitals (Table 3). High follow-up hospitals had the lowest rates of all postdischarge outcomes but had similar Centers for Medicare & Medicaid Services star ratings and patient experience scores compared with medium and low follow-up hospitals (eTable 5 in the Supplement).

## Discussion

In this study of 9 470 626 ED visits from 2011 to 2016, we found that approximately 29% of Medicare beneficiaries aged 65 years and older did not have an ambulatory follow-up visit within 30 days of ED discharge, while nearly 60% lacked follow-up with 1 week. Beneficiary Medicaid eligibility, Black race, and treatment at a rural hospital were associated with a lower rates of follow-up after ED discharge. Ambulatory follow-up was associated with approximately half the risk of 30-day postdischarge mortality but a 22% higher hazard of hospitalization. This association was present across individual conditions and hospitals but was strongest for those hospitals with the highest rates of follow-up after ED discharge, which tended to have better postdischarge outcomes in general. Additionally, these associations were strongest for conditions with the greatest risk of death after ED discharge in our sample.

Inconsistent rates of ambulatory follow-up after ED discharge among Medicare beneficiaries suggest an opportunity to improve care. Given the older age and greater chronic disease burden among Medicare beneficiaries relative to other ED patients, the immediate postdischarge period carries particularly high risk.^4^ Within this high-risk population, our analyses showed that vulnerable subgroups, such as those who were Medicaid eligible, had even longer times to follow-up, as did Black beneficiaries, for whom disparities in health care access and quality have been well-documented.^30^ Lower mortality among those with ambulatory follow-up suggests that access to follow-up care may be a key driver of postdischarge outcomes and a potential target for reducing health care disparities.^4^

Our results also underscore the potential trade-off between reducing acute care utilization and improving quality by reducing mortality.^31,32^ Beneficiaries with follow-up visits had a higher risk of a downstream inpatient hospitalization and lower mortality compared with those without follow-up. Clinicians caring for Medicare beneficiaries after ED discharge may be appropriately referring persistently ill patients back to the hospital to prevent further deterioration. Rather than representing failed transitions of care, this subset of readmissions may represent follow-up clinicians serving as a safety net in this postdischarge period. Similar phenomena have been suggested for patients discharged after an inpatient hospitalization.^31^ Taken together, these findings suggest that while avoiding an ED visit or hospitalization is desirable, a subset of acute visits may actually prevent mortality; thus, policies with a disproportionate focus on reducing acute care utilization could lead to unintended harm.

Our results are consistent with numerous studies documenting socioeconomic disparities in health care access and outcomes.^17,23,26,33,34,35^ While insurance type is an important determinant of access, we observed disproportionately lower rates of follow-up among non-White and Medicaid-eligible beneficiaries, even among traditional Medicare beneficiaries.^12,17^ Several factors likely contribute to these disparities, including racial discrimination in scheduling appointments, barriers to accessing nonemergency transport, and limited Medicaid expansion, less generous benefits, and a higher threshold for eligibility in some rural states.^36,37,38^ Among hospital characteristics, treatment at a rural ED has been shown to be associated with higher mortality rates among Medicare beneficiaries. Our findings are consistent with literature suggesting that barriers to ambulatory care access may be a key driver of poor health outcomes for Medicare beneficiaries treated in rural settings.^3,39,40^ These findings also serve as further evidence that hospitals serving populations in areas with disproportionately fewer resources may be penalized for factors beyond their control.^34,41^

### Limitations

Our study has a number of limitations. Findings among Medicare beneficiaries aged 65 years and older may not be generalizable to younger, healthier individuals with lower risk for mortality or readmission.^42^ However, Medicare beneficiaries account for growing share of ED visits, and lessons may be generalizable given that 60% of US residents have at least 1 chronic condition.^2,43,44^ Our study is observational, and therefore, the associations suggest, but do not prove, the benefit of ambulatory follow-up after ED discharge. The observational nature of our analysis and inability to completely control for patients who died before completing follow-up should be considered in future policy recommendations. We considered whether the association between higher rates of ambulatory care follow-up and lower postdischarge mortality was confounded by healthier patients who have fewer barriers to follow-up by virtue of their better health and thus are also more likely to survive. However, it is also possible that the bias goes the other way and that sicker patients are more likely to seek postdischarge care, leading to an underestimate of the benefit of ambulatory follow-up. Either a randomized clinical trial or daily monitoring of patient severity would be required to avoid or totally control for confounding at the time of the ambulatory visit. In this study, we controlled for patient characteristics, diagnosis categories, and chronic conditions to try to reduce confounding. The magnitude of the adjusted association seems too large to be entirely explained by unmeasured differences in patient severity alone. The fact that patients who had ambulatory follow-up were also more likely to have a subsequent hospitalization argues against this hypothesis, given that healthier patients generally do not use more hospital care. Furthermore, our findings were similar among hospitals with low rates of follow-up, where patients are more likely to experience barriers to accessing care relative to hospitals with high follow-up rates.

## Conclusions

In this study of Medicare beneficiaries aged 65 years and older who were discharged from US EDs from 2011 to 2016, nearly one-third (29%) lacked an ambulatory follow-up visit within 30 days. There was substantial variation among patients and hospitals in rates of follow-up after ED discharge, with lower follow-up rates among Medicaid-eligible beneficiaries, Black beneficiaries, and those treated in rural EDs. Ambulatory follow-up was associated with lower risk of postdischarge mortality but higher risk of a subsequent inpatient hospitalization. This association was observed for hospitals with high, medium, and low rates of follow-up but was greatest among hospitals with the highest rates of ambulatory care access. These findings suggest return visits to the ED or inpatient setting may be life-saving for some patients and that excess reductions in postdischarge acute care utilization may contribute to avoidable harm. Ambulatory follow-up care after ED discharge may be an important driver of outcomes among Medicare beneficiaries discharged from the ED.

## Notes

Supplement. **eTable 1.** Proportion of Visits by Provider Specialty for the 20 Most Frequent Specialties **eTable 2.** Association Between Ambulatory Follow-up and Risk of Postdischarge Mortality, Emergency Department Visits, and Inpatient Stays Within 30 Days Among Medicare Beneficiaries Treated in the Emergency Department and Discharged From 2011 to 2016 **eTable 3.** Association Between Ambulatory Follow-up at 7 Days and Postdischarge Mortality, Subsequent Emergency Department Visit, and Inpatient Stay Among Medicare Beneficiaries Treated in the Emergency Department and Discharged From 2011 to 2016 **eTable 4.** Association Between Ambulatory Follow-up and Risk of 30-Day Postdischarge Events for Emergency Department Visits for the Highest-Risk Conditions Among Medicare Beneficiaries Discharged From 2011 to 2016 **eTable 5.** Variation in 30-Day Postdischarge Outcomes and Quality Measure Performance Among Hospitals With High, Medium, and Low Rates of Follow-up After Discharge Home From the Emergency Department From 2011 to 2016 **eAppendix.** Technical Appendix **eFigure.** Exclusion Criteria
