# Supplementary material for: Ambulatory Follow-up and Outcomes Among Medicare Beneficiaries After Emergency Department Discharge
Source: JAMA Netw Open. 2020 Oct 9;3(10):e2019878. doi: 10.1001/jamanetworkopen.2020.19878 (PMC7547366; doi:10.1001/jamanetworkopen.2020.19878)
Supplement: Supplement. — eTable 1. Proportion of Visits by Provider Specialty for the 20 Most Frequent Specialties eTable 2. Association Between Ambulatory Follow-up and Risk of Postdischarge Mortality, Emergency Department Visits, and Inpatient Stays Within 30 Days Among Medicare Beneficiaries Treated in the Emergency Department and Discharged From 2011 to 2016 eTable 3. Association Between Ambulatory Follow-up at 7 Days and Postdischarge Mortality, Subsequent Emergency Department Visit, and Inpatient Stay Among Medicare Beneficiaries Treated in the Emergency Department and Discharged From 2011 to 2016 eTable 4. Association Between Ambulatory Follow-up and Risk of 30-Day Postdischarge Events for Emergency Department Visits for the Highest-Risk Conditions Among Medicare Beneficiaries Discharged From 2011 to 2016 eTable 5. Variation in 30-Day Postdischarge Outcomes and Quality Measure Performance Among Hospitals With High, Medium, and Low Rates of Follow-up After Discharge Home From the Emergency Department From 2011 to 2016 eAppendix. Technical Appendix eFigure. Exclusion Criteria [file jamanetwopen-e2019878-s001.pdf]

Supplemental Online Content

Lin MP, Burke RC, Orav EJ, Friend TH, Burke LG. Ambulatory follow-up and outcomes among Medicare beneficiaries after emergency department discharge. *JAMA Netw Open*. 2020;3(10):e2019878. doi:10.1001/jamanetworkopen.2020.19878

eTable 1. Proportion of Visits by Provider Specialty for the 20 Most Frequent Specialties
eTable 2. Association Between Ambulatory Follow-up and Risk of Postdischarge Mortality, Emergency Department Visits, and Inpatient Stays Within 30 Days Among Medicare Beneficiaries Treated in the Emergency Department and Discharged From 2011 to 2016
eTable 3. Association Between Ambulatory Follow-up at 7 Days and Postdischarge Mortality, Subsequent Emergency Department Visit, and Inpatient Stay Among Medicare Beneficiaries Treated in the Emergency Department and Discharged From 2011 to 2016
eTable 4. Association Between Ambulatory Follow-up and Risk of 30-Day Postdischarge Events for Emergency Department Visits for the Highest-Risk Conditions Among Medicare Beneficiaries Discharged From 2011 to 2016
eTable 5. Variation in 30-Day Postdischarge Outcomes and Quality Measure Performance Among Hospitals With High, Medium, and Low Rates of Follow-up After Discharge Home From the Emergency Department From 2011 to 2016
eAppendix. Technical Appendix
eFigure. Exclusion Criteria

This supplemental material has been provided by the authors to give readers additional information about their work.

© 2020 Lin MP et al. *JAMA Network Open.*

eTable 1. Proportion of Visits by Provider Specialty for the 20 Most Frequent Specialties

Specialty Description	N	%
Internal medicine	1,433,515	21.5%
Family practice	1,296,128	19.5%
Cardiology	427,666	6.6%
Orthopedic surgery	404,751	6.1%
Nurse practitioner	333,927	5.0%
Urology	278,888	4.2%
Ophthalmology	255,545	3.8%
Physician assistant	216,582	3.3%
Otolaryngology	137,037	2.1%
Podiatry	132,535	2.0%
Hematology/oncology	131,811	2.0%
Gastroenterology	121,352	1.8%
Neurology	115,591	1.7%
General surgery	109,666	1.6%
Optometrist	99,499	1.5%
Dermatology	97,311	1.5%
Pulmonary disease	95,477	1.4%
General practice	69,724	1.0%
Nephrology	61,689	0.9%
Psychiatry	56,962	0.9%

*Primary care specialties were defined as internal medicine, family practice, general practice and geriatric medicine and accounted for 42.4% of the follow-up visits in this sample.

© 2020 Lin MP et al. *JAMA Network Open.*

eTable 2. Association Between Ambulatory Follow-up and Risk of Postdischarge Mortality, Emergency Department Visits, and Inpatient Stays Within 30 Days Among Medicare Beneficiaries Treated in the Emergency Department and Discharged From 2011 to 2016[a]

		Mortality Hazard Ratio[b] (95% CI)	ED Visits Hazard Ratio[c] (95% CI)	Inpatient Stay Hazard Ratio[c] (95% CI)
**Ambulatory follow-up as a time-varying covariate**		0.49 (0.49-0.50)	1.01 (1.00-1.01)	1.22 (1.21-1.23)
**Time (years)**		0.995 (0.992-0.999)	1.013 (1.012-1.014)	0.961 (0.960-0.962)
**Age (years)**		1.039 (1.039-1.040)	0.994 (0.993-0.994)	0.997 (0.997-0.997)
**Sex**	Female	**REF**	**REF**	**REF**
	Male	1.35 (1.33-1.37)	1.070 (1.066-1.074)	1.00 (1.00-1.01)
**Race**	White	**REF**	**REF**	**REF**
	Black	1.35 (1.33-1.37)	1.17 (1.16-1.17)	0.95 (0.94-0.96)
	Hispanic	0.73 (0.69-0.76)	0.94 (0.93-0.95)	0.90 (0.88-0.91)
	Asian	0.89 (0.85-0.95)	0.83 (0.81-0.84)	0.87 (0.85-0.89)
	North American Native	0.99 (0.93-1.07)	1.11 (1.09-1.13)	1.05 (1.02-1.08)
	Other	0.95 (0.90-1.10)	0.93 (0.92-0.95)	0.97 (0.95-0.99)
	Unknown	0.91 (0.82-1.01)	0.86 (0.84-0.89)	0.93 (0.89-0.96)
**Medicaid Eligible**	No	**REF**	**REF**	**REF**
	Yes	1.35 (1.33-1.37)	1.28 (1.28-1.29)	0.97 (0.96-0.97)
**Principal Diagnosis**	Minor injuries	1.10 (1.04-1.17)	0.62 (0.61-0.63)	1.19 (1.17-1.22)
	Major injuries	1.56 (1.42-1.71)	0.66 (0.64-0.68)	1.07 (1.03-1.12)
	Other injuries	1.05 (1.00-1.11)	0.68 (0.68-0.69)	0.93 (0.91-0.95)
	Abdominal pain	1.22 (1.14-1.30)	0.94 (0.93-0.95)	1.43 (1.40-1.46)
	Chest pain	0.79 (0.74-0.85)	0.69 (0.68-0.70)	1.01 (0.99-1.03)
	Dizziness, vertigo and syncope	0.55 (0.51-0.58)	0.55 (0.54-0.55)	0.89 (0.86-0.91)
	Headache	0.64 (0.58-0.70)	0.97 (0.96-0.99)	1.04 (1.01-1.07)
	Other symptoms	1.46 (1.38-1.55)	0.78 (0.77-0.79)	1.38 (1.35-1.41)
	Upper respiratory infections	0.58 (0.54-0.62)	0.70 (0.69-0.71)	1.01 (0.98-1.03)
	Intestinal infections	0.97 (0.82-1.15)	0.73 (0.70-0.76)	1.53 (1.45-1.61)
	Urinary tract infection	1.22 (1.15-1.29)	0.89 (0.87-0.90)	1.39 (1.36-1.42)
	Other infectious and parasitic diseases	1.28 (1.18-1.39)	1.00 (0.98-1.02)	1.20 (1.17-1.24)
	Skin and subcutaneous infection	0.68 (0.63-0.74)	1.08 (1.07-1.10)	1.31 (1.28-1.34)
	Endocrine, nutritional; immunity and metabolic disorders	2.23 (2.10-2.37)	0.75 (0.73-0.76)	1.35 (1.32-1.39)
	Diabetes mellitus	1.21 (1.12-1.30)	0.74 (0.72-0.75)	1.17 (1.14-1.20)
	Hypertension	0.62 (0.57-0.67)	0.85 (0.83-0.86)	0.95 (0.93-0.98)
	Nonatherosclerotic heart disease	4.09 (3.63-4.60)	0.82 (0.78-0.87)	1.66 (1.55-1.77)
	Dysrhythmias	0.99 (0.93-1.07)	0.73 (0.71-0.74)	1.16 (1.13-1.19)
	Ischemic heart disease	2.05 (1.85-2.28)	0.67 (0.65-0.70)	1.54 (1.47-1.61)
	Congestive heart failure	2.25 (2.11-2.40)	0.74 (0.72-0.75)	1.55 (1.51-1.59)
	Circulatory disorders	1.54 (1.44-1.64)	0.69 (0.68-0.71)	1.31 (1.27-1.34)
	Cerebrovascular disease	2.35 (2.17-2.53)	0.58 (0.57-0.60)	1.35 (1.30-1.40)

© 2020 Lin MP et al. *JAMA Network Open.*

		Mortality Hazard Ratio[b] (95% CI)	ED Visits Hazard Ratio[c] (95% CI)	Inpatient Stay Hazard Ratio[c] (95% CI)
	Diseases of the blood	2.37 (2.21-2.54)	0.78 (0.76-0.80)	1.48 (1.44-1.54)
	Neoplasms	7.82 (7.34-8.32)	0.98 (0.95-1.00)	2.31 (2.24-2.39)
	Mental illness	1.47 (1.38-1.57)	1.02 (1.01-1.04)	1.26 (1.23-1.29)
	Nervous system disorders	1.15 (1.08-1.22)	0.78 (0.77-0.79)	1.10 (1.07-1.12)
	Pneumonia	2.06 (1.93-2.20)	0.71 (0.70-0.72)	1.55 (1.51-1.59)
	Other respiratory disease	1.59 (1.50-1.68)	0.92 (0.91-0.93)	1.16 (1.14-1.19)
	Chronic obstructive pulmonary disease	1.24 (1.17-1.32)	0.77 (0.76-0.78)	1.45 (1.42-1.48)
	Asthma	0.84 (0.74-0.95)	0.82 (0.80-0.84)	1.32 (1.28-1.37)
	Noninfectious lung disease	3.62 (3.34-3.91)	0.77 (0.75-0.80)	1.90 (1.83-1.98)
	Gastrointestinal system diseases	1.31 (1.24-1.39)	0.83 (0.82-0.84)	1.40 (1.38-1.44)
	Other renal and genito-urinary diseases	1.08 (1.02-1.15)	1.13 (1.11-1.14)	1.34 (1.31-1.37)
	Renal disease	3.37 (3.09-3.66)	0.80 (0.77-0.83)	1.50 (1.44-1.57)
	Pregnancy- and childbirth-related disorders	0.00 (0.00-3.08 x 10^{94})	1.83 (0.63-5.32)	0.02 (0.01-0.06)
	Diseases of the musculoskeletal system	1.03 (0.97-1.09)	0.82 (0.81-0.83)	1.25 (1.23-1.28)
	Complications and adverse events	1.53 (1.44-1.63)	0.99 (0.98-1.01)	1.43 (1.39-1.46)
	Other residual codes	**REF**	**REF**	**REF**
**Chronic Conditions**	Alzheimer's/Dementia	1.71 (1.69-1.73)	1.22 (1.22-1.23)	1.29 (1.28-1.29)
	Acute Myocardial Infarction	1.62 (1.58-1.66)	1.13 (1.12-1.14)	1.60 (1.59-1.62)
	Anemia	1.47 (1.45-1.49)	1.17 (1.16-1.17)	1.88 (1.87-1.89)
	Asthma	0.71 (0.69-0.72)	1.16 (1.15-1.16)	1.11 (1.10-1.12)
	Atrial Fibrillation	0.95 (0.94-0.97)	1.06 (1.05-1.06)	1.24 (1.23-1.25)
	Cataract	0.45 (0.44-0.46)	0.939 (0.935-0.943)	0.86 (0.86-0.87)
	Congestive Heart Failure	1.71 (1.69-1.73)	1.080 (1.076-1.084)	1.34 (1.33-1.34)
	Chronic Kidney Disease	1.84 (1.82-1.87)	1.08 (1.08-1.09)	1.62 (1.61-1.63)
	Breast Cancer	1.19 (1.16-1.22)	1.07 (1.06-1.07)	1.19 (1.17-1.20)
	Colorectal Cancer	1.36 (1.32-1.39)	1.10 (1.10-1.11)	1.37 (1.26-1.39)
	Endometrial Cancer	1.41 (1.33-1.50)	1.14 (1.12-1.16)	1.50 (1.47-1.53)
	Lung Cancer	3.66 (3.59-3.74)	1.12 (1.11-1.13)	1.57 (1.56-1.59)
	Prostate Cancer	0.99 (0.97-1.02)	1.10 (1.09-1.11)	1.17 (1.16-1.18)
	COPD	1.24 (1.23-1.26)	1.21 (1.21-1.22)	1.38 (1.37-1.39)
	Depression	0.92 (0.91-0.94)	1.31 (1.31-1.32)	1.27 (1.26-1.28)
	Diabetes	1.03 (1.01-1.04)	0.98 (0.97-0.98)	1.00 (0.99-1.00)
	Glaucoma	0.54 (0.52-0.55)	0.950 (0.945-0.954)	0.86 (0.85-0.86)
	Hip Fracture	1.08 (1.05-1.11)	1.00 (0.99-1.01)	1.53 (1.52-1.55)
	Hyperlipidemia	0.59 (0.58-0.60)	0.98 (0.98-0.99)	1.08 (1.07-1.08)
	Benign Prostatic Hypertrophy	0.79 (0.78-0.81)	1.22 (1.21-1.23)	1.29 (1.28-1.30)
	Hypertension	0.70 (0.69-0.72)	1.26 (1.26-1.27)	1.46 (1.45-1.47)
	Hypothyroidism	0.89 (0.87-0.90)	1.04 (1.04-1.05)	1.08 (1.08-1.09)
	Ischemic Heart Disease	1.11 (1.09-1.12)	1.13 (1.13-1.13)	1.10 (1.09-1.10)
	Osteoporosis	0.93 (0.91-0.95)	1.10 (1.09-1.10)	1.27 (1.27-1.28)
	Rheumatoid Arthritis/Osteoarthritis	0.75 (0.74-0.75)	1.17 (1.16-1.17)	1.06 (1.06-1.07)

© 2020 Lin MP et al. *JAMA Network Open.*

		Mortality Hazard Ratio[b] (95% CI)	ED Visits Hazard Ratio[c] (95% CI)	Inpatient Stay Hazard Ratio[c] (95% CI)
	Stroke/Transient Ischemic Attack	1.15 (1.13-1.17)	1.14 (1.14-1.15)	1.38 (1.37-1.39)

[a]Cox proportional hazards models with the time to each post-discharge event as the outcome and ambulatory follow-up as a time-varying covariate as the primary predictor. We incorporated beneficiary age, sex, race, Medicaid eligibility, year of the visit, principal diagnosis category and beneficiary chronic conditions as covariates. We adjusted for clustering by hospital. [b]Hazard ratio less than one means a longer time until the outcome event. [c]For the outcomes of ED visits and inpatient stays, we also incorporated mortality as a competing risk. Outcomes were censored at 30 days.

© 2020 Lin MP et al. *JAMA Network Open.*

eTable 3. Association Between Ambulatory Follow-up at 7 Days and Postdischarge Mortality, Subsequent Emergency Department Visit, and Inpatient Stay Among Medicare Beneficiaries Treated in the Emergency Department and Discharged From 2011 to 2016[a]

	Hazard Ratio[b] (95% CI)	P-Value
**Mortality**	0.44 (0.42 to 0.46)	<.001
**ED Return**	0.96 (0.95 to 0.96)	<.001
**Inpatient Stay**	1.14 (1.13 to 1.15)	<.001

[a]Cox proportional hazards models with the time to each post-discharge event as the outcome and ambulatory follow-up as a time-varying covariate as the primary predictor. We incorporated beneficiary age, sex, race, Medicaid eligibility, year of the visit, principal diagnosis category and beneficiary chronic conditions as covariates. We adjusted for clustering by hospital. [b]Hazard ratio less than one means a longer time until the outcome event. [c]For the outcomes of ED visits and inpatient stays, we also incorporated mortality as a competing risk. Outcomes were censored at 7 days.

© 2020 Lin MP et al. *JAMA Network Open.*

eTable 4. Association Between Ambulatory Follow-up and Risk of 30-Day Postdischarge Events for Emergency Department Visits for the Highest-Risk Conditions Among Medicare Beneficiaries Discharged From 2011 to 2016[a]

	N (%)	Proportion of Patients with Ambulatory Follow-Up At:		Hazard Ratio (95% CI)[b] for the Effect of an Ambulatory Visit On:		
		7 Days	30 Days	Mortality	ED Return	Inpatient Stay
**All Conditions**	**9,470,626 (100%)**	40.5%	70.8%	0.49 (0.49-0.50)	1.01 (1.00-1.03)	1.22 (1.21-1.23)
**Cerebrovascular disease**	43,438 (0.5%)	46.3%	72.9%	0.23 (0.20-0.28)	1.08 (1.02-1.15)	1.30 (1.20-1.40)
**Neoplasms**	29,806 (0.3%)	46.1%	75.7%	0.37 (0.34-0.41)	1.25 (1.17-1.32)	1.56 (1.47-1.66)
**Renal disease**	13,473 (0.1%)	35.2%	65.3%	0.38 (0.31-0.48)	0.99 (0.90-1.09)	1.13 (1.03-1.25)
**Pneumonia**	87,119 (0.9%)	40.7%	70.3%	0.41 (0.37-0.46)	0.92 (0.88-0.96)	1.08 (1.03-1.13)
**Ischemic heart disease**	18,450 (0.2%)	39.6%	75.3%	0.42 (0.30-0.58)	1.16 (1.04-1.24)	1.16 (1.04-1.29)
**Congestive heart failure**	60,687 (0.6%)	46.6%	74.9%	0.43 (0.39-0.47)	1.03 (0.99-1.07)	1.04 (1.00-1.09)
**Nonatherosclerotic heart disease**	6,950 (0.1%)	47.4%	77.6%	0.44 (0.28-0.67)	1.04 (0.89-1.22)	1.04 (0.87-1.25)
**Endocrine, nutritional, metabolic**	195,620 (2.1%)	38.7%	68.9%	0.49 (0.46-0.52)	1.07 (1.04-1.09)	1.17 (1.13-1.20)
**Diseases of the blood**	43,821 (0.5%)	41.3%	72.5%	0.50 (0.45-0.56)	1.02 (0.97-1.07)	1.18 (1.13-1.25)
**Noninfectious lung disease**	18,528 (0.2%)	44.8%	74.4%	0.53 (0.45-0.62)	1.01 (0.92-1.10)	1.40 (1.29-1.52)

[a]Cox proportional hazards models with the time to each post-discharge event as the outcome and ambulatory follow-up as a time-varying covariate as the primary predictor. We incorporated beneficiary age, sex, race, Medicaid eligibility, year of the visit, principal diagnosis category and beneficiary chronic conditions as covariates. We adjusted for clustering by hospital. [b]Hazard ratio less than one means a longer time until the outcome event. [c]For the outcomes of ED visits and inpatient stays, we also incorporated mortality as a competing risk. Outcomes were censored at 30 days.

© 2020 Lin MP et al. *JAMA Network Open.*

eTable 5. Variation in 30-Day Postdischarge Outcomes[a] and Quality Measure Performance[b] Among Hospitals With High, Medium, and Low Rates of Follow-up[c] After Discharge Home From the Emergency Department From 2011 to 2016

	Adjusted Mortality[a]	Adjusted ED Revisit[a]	Adjusted IP Visit[a]	Mean CMS Star Rating[b,d]	Mean Patient Experience Rating[b,e]
**Low Follow-Up Hospitals[d,f]**	1.49%	22.2%	11.1%	3.12	72.3%
**Medium Follow-Up Hospitals**	1.41%	18.2%	9.7%	2.97	70.3%
**High Follow-Up Hospitals**	1.28%	16.7%	9.3%	3.13	71.5%

[a]Linear probability models with the time to each post-discharge event as the outcome and ambulatory follow-up as a time-varying covariate as the primary predictor. We incorporated beneficiary age, sex, race, Medicaid eligibility, year of the visit, principal diagnosis category and beneficiary chronic conditions as covariates. We adjusted for clustering by hospital. [b]Publicly-reported quality measures in the Centers for Medicare and Medicare and Medicaid Services (CMS) Hospital Compare program. [c] Adjusted rates of ambulatory follow-up after discharge from the ED were determined from a linear probability model adjusted for the same set of covariates. d Overall hospital star rating for each hospital was downloaded directly from the Hospital Compare website for the 2015-2016 time period. [e] We used the percentage of patients that gave the hospital a high rating for patient experience (measure ID H_HSP_RATING_9_10) for the 2015-2016 time period. [f] Low-follow-up hospitals were those in the lowest quartile with respect to adjusted rates of ambulatory follow-up, high follow-up hospitals were those in the top quartile of ambulatory follow-up rates and the remaining hospitals were considered medium follow-up hospitals.

© 2020 Lin MP et al. *JAMA Network Open.*

eAppendix. Technical Appendix

We included traditional Medicare beneficiaries age 65 and older from 2011-2016. We excluded beneficiaries who lacked Part B coverage (only 0.3% of traditional Medicare beneficiaries during our study years). Additional exclusions are outlined in eFigure1 below.

© 2020 Lin MP et al. *JAMA Network Open.*

eFigure. Exclusion Criteria

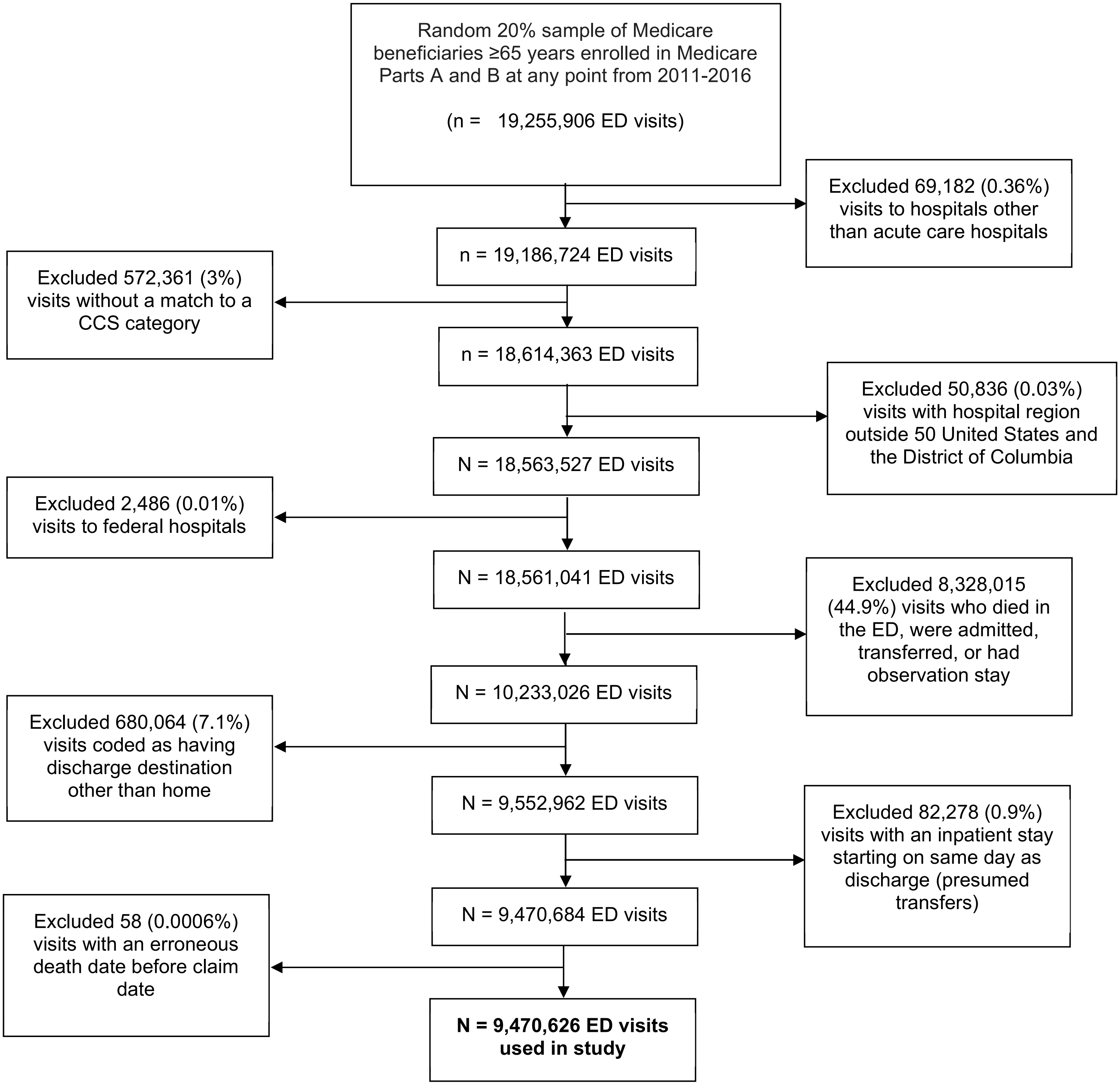

© 2020 Lin MP et al. *JAMA Network Open.*

We included only outpatient visits with a discharge status Code of 1 (discharge to home).

Identifying ambulatory follow-up visits

Included visits for the following BETOS codes:
M1A Office Visits-New
M1B Office Visits - Established
M5B Specialist - Psychiatry
M5C Specialist - Ophthalmology
M5D Specialist - Other
M6 Consultations
M4A Home Visit

We excluded visits for the following BETOS codes:
M2A Hospital Visit - Initial
M2B Hospital Visit - Subsequent
M2C Hospital Visit – Critical Care
M3 Emergency Room Visit
M4B Nursing Home Visit
M5A Specialist-Pathology

We also excluded ambulatory follow-up visits with the following place of services codes on the carrier claim:
1 (Pharmacy)
17 (Walk-in/Retail Health)
20 (Urgent Care)
21 (Inpatient Hospital)
23 (Emergency Room-Hospital)
31 (SNF)
32 (Nursing Facility)
51 (Inpatient Psych)

Categorizing Visit Diagnosis
We categorized ED visits first by identifying the principal diagnosis and categorized International Classification of Diseases (ICD) ninth or tenth revision codes into Healthcare Cost and Utilization Project Clinical Classification Software (HCUP-CCS) categories. We aggregated these categories into the following 38 categories as shown below based on a method previously described for studying emergency department visits (Gabayan et al. Ann Emerg Med. 2011 Dec;58(6):551-558.e2. doi: 10.1016/j.annemergmed.2011.07.001.) according to the following crosswalk:

Category	Definition	Multilevel CCS Codes	CCS Code Description
1	Minor injuries	16.1, 16.2, 16.7	Sprains, fractures, and joint disorders
2	Major injuries	16.3, 16.4, 16.5	Spinal cord, intracranial, crushing/internal organ injury
3	Other injuries	16.6, 16.8, 16.9, 16.11, 16.12	Including burns, wounds, poisonings, superficial injuries
4	Symptoms: abdominal pain	17.1.7	Abdominal pain
5	Symptoms: chest pain	7.2.5	Chest pain
6	Symptoms: dizziness, vertigo and syncope	6.8.2, 17.1.1	Dizziness, vertigo, and syncope
7	Symptoms: headache	6.5	Headache
8	Other symptoms	17.1.2, 17.1.3, 17.1.4, 17.1.5, 17.1.6, 17.1.8, 17.1.9	Other symptoms, signs, and ill-defined conditions
9	Upper respiratory infections	8.1.2, 8.1.3, 8.1.4, 8.1.5	Upper respiratory infections excluding pneumonia

© 2020 Lin MP et al. *JAMA Network Open.*

10	Intestinal infections	9.1	Intestinal infections
11	Urinary tract infection	10.1.4	Urinary tract infection and symptoms
12	Other infectious and parasitic diseases	1, 6.1, 13.1	Other infectious and parasitic diseases, meningitis, infective arthritis, bacterial, mycoses, viral
13	Skin and subcutaneous infection	12.1	Skin and subQ infection
14	Endocrine, nutritional; immunity and metabolic disorders	3.1, 3.4, 3.5, 3.6, 3.7, 3.8, 3.9, 3.10, 3.11	Endocrine diseases; nutritional and metabolic diseases; immunity disorders
15	Diabetes mellitus	3.2, 3.3	Diabetes with and without complications
16	Hypertension	7.1	Hypertension
17	Nonatherosclerotic heart disease	7.2.1, 7.2.2, 7.2.6, 7.2.7, 7.2.10	Valvular heart disease, myocarditis, pericarditis
18	Dysrhythmias	7.2.8, 7.2.9	Dysrhythmias and conduction disorders
19	Ischemic heart disease	7.2.3, 7.2.4	Ischemic heart disease and myocardial infarction
20	Congestive heart failure	7.2.11	Congestive heart failure
21	Circulatory disorders	7.4, 7.5	Diseases of arteries, arterioles, veins, lymphatics, and capillaries
22	Cerebrovascular disease	7.3	Cerebrovascular disease
23	Diseases of the blood	4	Diseases of the blood and blood-forming organs
24	Neoplasms	2	Neoplasms
25	Mental illness	5	Mental illness
26	Nervous system disorders	6.2, 6.3, 6.4, 6.6, 6.7, 6.8.1, 6.8.3, 6.9	Nervous system disorders
27	Pneumonia	8.1.1	Pneumonia
28	Other respiratory disease	8.6, 8.7, 8.8, 8.9	Respiratory insufficiency, lung disease caused by external agents, other lower and upper respiratory diseases
29	Chronic obstructive pulmonary disease	8.2	Chronic obstructive pulmonary disease
30	Asthma	8.3	Asthma
31	Noninfectious lung disease	8.4, 8.5	Pleurisy, pneumothorax, pneumonitis
32	Gastrointestinal system diseases	9.2, 9.3, 9.4, 9.5, 9.6, 9.7, 9.8, 9.9, 9.10, 9.11, 9.12	Disorders of mouth, esophagus, and upper gastrointestinal tract; abdominal hernia; lower gastrointestinal disorders, biliary, liver, and pancreatic tree disorders; gastrointestinal hemorrhage; noninfectious gastroenteritis
33	Other renal and genito-urinary diseases	10.1.5, 10.1.6, 10.1.7, 10.2, 10.3, 10.1.8	Renal or urinary tract calculus; other diseases of bladder and urethra; diseases of male and female genital organs; genito-urinary symptoms

© 2020 Lin MP et al. *JAMA Network Open.*

34	Renal disease	10.1.1, 10.1.2, 10.1.3	Nephritis, nephrosis, renal sclerosis; acute renal failure; chronic renal failure
35	Pregnancy- and childbirth-related disorders	11	Pregnancy- and childbirth-related disorders
36	Diseases of the musculoskeletal system, skin, and connective tissue	12.2, 12.3, 12.4, 13.2, 13.3, 13.4, 13.5, 13.6, 13.7, 13.8, 13.9	Sprains, fractures, and joint disorders
37	Complications and adverse events	16.10	Spinal cord, intracranial, crushing/internal organ injury
38	Other residual codes	14, 15, 17.2, 18	Including burns, wounds, poisonings, superficial injuries

Of note, for visits using ICD-10 codes, we used the Centers for Medicare and Medicaid Services (CMS) General Equivalence Mappings to convert all ICD-10 codes to ICD-9. We got this crosswalk from the National Bureau of Economic Research (https://data.nber.org/data/icd9-icd-10-cm-and-pcs-crosswalk-general-equivalence-mapping.html), but CMS is the original source and they publish a new version each year.

We found that in most cases, an ICD-10 code maps directly to one ICD-9 code. There are some ICD-10 codes that have either have multiple approximate matches or should be represented by a set of codes, so we needed to develop logic to choose a single ICD-9 code, using flag variables that CMS provided- approximate, scenario, and choice list. The logic is summarized below.

1. If there is an exact match, keep that over an approximate match (approximate=0)
2. When there should be multiple codes to represent an ICD-10 code, keep the ICD-9 code that is listed first within the scenario (choice_list=1).
3. If there are still multiple codes after the above steps, choose the most common ICD-9 code, based on the count for that code in 2014 (the most recent full year of ICD-9 data).

Hospital Characteristics

The Following Hospital Characteristics were identified from the 2014 American Hospital Association Annual Survey:

Size- Hospital size was determined based on the number of hospital beds. Hospitals with 1-99 beds were considered small hospitals, medium hospitals were those with 100-399 beds and large hospitals had 400 or more beds

Region- Northeast, Midwest, South and West (other regions were excluded).

Control Type- For-Profit, Not-for-Profit, Government, Non-Federal

Urban/Rural location- This was defined by the Core Based Statistical Area (CBSA). CBSA type of metropolitan or micropolitan was considered urban while rural CBSA type was considered rural.

Teaching Status- Major teaching hospitals were defined as those with membership in the Council of Teaching Hospitals (COTH), minor teaching hospitals were those without COTH membership but that reported a medical school affiliation to the American Medical Association. All other hospitals were designated as non-teaching.

Hospital safety-net status was defined using the Medicare Impact File disproportionate share patient percentage. This percentage measures the degree to which hospitals serve Medicaid beneficiaries and the uninsured. Consistent with prior literature (Figueroa et al: *Med Care* 2017;55: 229–235), we considered hospitals in the highest quartile of

© 2020 Lin MP et al. *JAMA Network Open.*

disproportionate share percentage to be safety-net hospitals while all others were considered to be non-safety net hospitals

Hospital Compare Quality Measures

We downloaded data on Hospital Compare for the years 2015-2016

Overall Star Rating (1-5)

And percentage of patients who gave a rating of "9" or "10" (high) (Measure ID H_HSP_RATING_9_10)

© 2020 Lin MP et al. *JAMA Network Open.*
